# Default Mode Network Complexity and Cognitive Decline in Mild Alzheimer’s Disease

**DOI:** 10.3389/fnins.2018.00770

**Published:** 2018-10-23

**Authors:** Matthias Grieder, Danny J. J. Wang, Thomas Dierks, Lars-Olof Wahlund, Kay Jann

**Affiliations:** ^1^Translational Research Center, University Hospital of Psychiatry, University of Bern, Bern, Switzerland; ^2^USC Stevens Neuroimaging and Informatics Institute, Keck School of Medicine of USC, University of Southern California, Los Angeles, CA, United States; ^3^Division of Clinical Geriatrics, Department of Neurobiology, Care Sciences and Society, NVS, Karolinska Institute, Stockholm, Sweden

**Keywords:** multi-scale entropy, complexity, functional connectivity, resting-state fMRI, default mode network, Alzheimer’s disease, cognitive decline

## Abstract

The human resting-state is characterized by spatially coherent brain activity at a low temporal frequency. The default mode network (DMN), one of so-called resting-state networks, has been associated with cognitive processes that are directed toward the self, such as introspection and autobiographic memory. The DMN’s integrity appears to be crucial for mental health. For example, patients with Alzheimer’s disease or other psychiatric conditions show disruptions of functional connectivity within the brain regions of the DMN. However, in prodromal or early stages of Alzheimer’s disease, physiological alterations are sometimes elusive, despite manifested cognitive impairment. While functional connectivity assesses the signal correlation between brain areas, multi-scale entropy (MSE) measures the complexity of the blood-oxygen level dependent signal within an area and thus might show local changes before connectivity is affected. Hence, we investigated alterations of functional connectivity and MSE within the DMN in fifteen mild Alzheimer’s disease patients as compared to fourteen controls. Potential associations of MSE with functional connectivity and cognitive abilities [i.e., mini-mental state examination (MMSE)] were assessed. A moderate decrease of DMN functional connectivity between posterior cingulate cortex and right hippocampus in Alzheimer’s disease was found, whereas no differences were evident for whole-network functional connectivity. In contrast, the Alzheimer’s disease group yielded lower global DMN-MSE than the control group. The most pronounced regional effects were localized in left and right hippocampi, and this was true for most scales. Moreover, MSE significantly correlated with functional connectivity, and DMN-MSE correlated positively with the MMSE in Alzheimer’s disease. Most interestingly, the right hippocampal MSE was positively associated with semantic memory performance. Thus, our results suggested that cognitive decline in Alzheimer’s disease is reflected by decreased signal complexity in DMN nodes, which might further lead to disrupted DMN functional connectivity. Additionally, altered entropy in Alzheimer’s disease found in the majority of the scales indicated a disturbance of both local information processing and information transfer between distal areas. Conclusively, a loss of nodal signal complexity potentially impairs synchronization across nodes and thus preempts functional connectivity changes. MSE presents a putative functional marker for cognitive decline that might be more sensitive than functional connectivity alone.

## Introduction

Brain activity during the human resting-state shows spatially coherent patterns at a low temporal frequency (Biswal et al., 1995; Raichle et al., 2001). Blood-oxygen level dependent (BOLD) resting-state functional MRI (rs-fMRI) revealed these so-called resting state networks (RSN), consisting of spatially segregated brain regions that are intrinsically co-activated and deactivated across time. The idea is widely accepted that these highly correlated brain regions are functionally connected, and the connectivity strength is represented by the correlation coefficient between given areas. Moreover, it has been shown that the RSNs or the functional connectivity (FC) are altered in subgroups of patients. In particular, FC appears to be related to disease severity or cognitive decline in dementia and normal aging (Petrella et al., 2011; Agosta et al., 2012; Brier et al., 2012).

The most widely studied RSN is the default mode network (DMN), which has been associated with mind wandering, autobiographic memory, future thinking, and introspection (for review, see Buckner and Carroll, 2007; Buckner et al., 2008). The core brain regions of the DMN are medial prefrontal cortex (MPFC), posterior cingulate cortex (PCC), left and right inferior parietal lobes (IPL), and left and right hippocampi (Hipp, Buckner et al., 2008). The DMN’s integrity appears to play an important role for the health of mind, since DMN disruptions have been reported in schizophrenia spectrum disorder (Bluhm et al., 2007; Garrity et al., 2007), depression (Wise et al., 2017), autism (Padmanabhan et al., 2017; Hogeveen et al., 2018), and Alzheimer’s disease (Jones et al., 2011; Cha et al., 2013). Specifically in Alzheimer’s disease, disease progression severity has been associated with reduced DMN FC, as compared to age-matched controls (Lustig et al., 2003; Greicius et al., 2004; Zhang et al., 2010; Zhou et al., 2010). Furthermore, the temporal anti-correlation between task-positive (i.e., RSNs resembling functional networks engaged during task execution) and the task-negative RSN (increased activity in absence of a task, i.e., DMN), normally found in healthy subjects, is attenuated in progressed stages of AD (Fox et al., 2005; Weiler et al., 2017). This inability to switch between the task-positive RSNs and the DMN is hypothesized to be related to cognitive impairment in AD. Moreover, Koch et al. (2012) have corroborated the DMN’s relevance in AD by demonstrating the diagnostic power of DMN connectivity strength to separate AD from healthy controls, while the prediction of mild cognitive impairment (MCI) was less obvious.

Despite the prospects of RSN-FC as a marker of cognitive decline, FC is an average measure of correlation between brain areas during a few minutes of fMRI scanning. FC has limited capability in characterizing the dynamic reorganization and regional activity of complex brain networks. Hence, assessing the dynamic properties of connections (network edges) between and within areas (network nodes) of the brain is a necessary step to further understand the normal brain function during resting state as well as putative disruptions of the functional organization in the course of a disease.

Non-linear statistical approaches have been applied for quantifying the regularity of biological signals such as approximate entropy (ApEn) or its variant sample entropy (SampEn; Pincus, 1991; Richman and Moorman, 2000; Liu et al., 2013; Sokunbi, 2014). When applied to several coarse-sampled scales from the original time series, SampEn can be extended to multi-scale entropy analysis (MSE; Costa et al., 2002). Smith et al. (2014) showed that healthy aging is associated with decreased MSE mainly in DMN regions such as middle temporal gyrus, MPFC, angular gyri, middle and superior medial frontal cortex, and Hipp. Smith et al. (2015) in a later study further showed that MCI is associated with reduced MSE in areas of the DMN. Such findings were confirmed by studies that reported a positive relationship between cognitive decline in familial AD and whole brain ApEn as well as regional entropy in precuneus, lateral parietal cortex, precentral gyrus, and paracentral gyrus (Liu et al., 2013; Wang et al., 2017). These preliminary studies suggest that MSE of rs-fMRI may provide a marker of cognitive decline in aging and dementia.

The purpose of the present study was to compare MSE of the DMN between a group of patients with mild AD and a group of age matched controls. We examined the added value of including a metric of regional (nodal) dynamics (MSE) to the inter-regional connectivity (edges) assessed with FC. We further tested the relation of MSE alteration and cognitive decline assessed by the mini-mental state examination (MMSE) as well as Boston Naming Task (BNT) scores. We expected generally lower MSE values in AD as compared to matched controls. More detailed hypotheses about regional DMN-MSE changes in AD were not made, since there is no comparable study available for hypothesis generation. However, we predicted a positive correlation between MSE and MMSE, which is in accordance with Liu et al. (2013). With focus on the standard FC analysis, we anticipated reduced FC in AD compared to HC, because most studies reported FC decreases in AD (Cha et al., 2013).

## Materials and Methods

### Participants

All participants provided written informed consent according to a protocol approved by the Regional Ethics Committee of Stockholm, Sweden, in accordance with the Declaration of Helsinki. Only native speakers of Swedish were included and exclusion criteria were the presence of medical or psychiatric disorders (other than dementia), intake of drugs affecting the nervous system, or any contraindications for MRI procedures. Fourteen healthy elderly control (HC) participants (aged 62–73 years) were included into data analysis after exclusion of one participant due to excessive movement artifacts in the MR-images. The mild Alzheimer’s disease (AD) group consisted of 15 patients (aged 53–83) after discarding two artifact-contaminated data-sets (Table 1). The patients were recruited at the Memory Clinic of the Geriatric Department at Karolinska University Hospital in Huddinge, Sweden. Hence, their diagnosis was performed by expert clinicians and were in accordance with the ICD-10 criteria. The patients with AD included in this study underwent a standard clinical procedure which consisted of examinations such as structural neuroimaging, lumbar puncture, blood analyses, and a neuropsychological assessment. Further inclusion criteria for all patients were a Global Deterioration Scale smaller than 6 (i.e., moderate dementia, or milder) and the Cornell Depression Scale below 8. Controls were screened with a neuropsychological test battery, comprised MMSE and BNT.

**Table 1:** Demographics and descriptive statistics.

	HC (*n* = 14)	AD (*n* = 15)	HC-AD	
	Mean (SD)	Mean (SD)	*U*	*p*-Value
Age, years	67.5 (3.5)	67.3 (8.6)	95.5	n.s.
Gender (F:M)	10:4	8:7		
Education, years	13.3 (3.0)	12.9 (3.0)	103.0	n.s.
MMSE (max 30)	28.8 (0.9)	25.0 (3.8)	16.5	<0.001
BNT (max 60)	53.7 (3.7)	45.8 (6.6)	30.5	0.001
GDS	n/a	2.9 (0.8)		
CDS	n/a	1.3 (1.2)		

*Group-wise statistics were performed using the non-parametric Mann–Whitney U-test.*

### Image Acquisition and Preprocessing

Data were acquired on a 3T Siemens Magnetom Trio MR Scanner (Siemens AG, Erlangen, Germany). GE EPI fMRI BOLD was recorded with 26 transversal slices; 3.0 × 3.0 in-plane and 4 mm slice thickness; TR/TE = 1600/35 ms, FA 90°; FoV = 240 × 240 mm; matrix = 92 × 92, 400 volumes acquired in 10 min 40 s. A structural T1-weighted MPRAGE was recorded with 176 sagittal slices, 0.9 × 0.9 in-plane and 1 mm slice thickness, TR/TE = 1900/2.57 ms; FoV = 230 × 230 mm; matrix = 256 × 256.

Preprocessing of fMRI data involved motion-realignment, linear drift correction (detrending), regression of motion (six motion parameters and first derivatives; Power et al., 2014) and physiological noise (WM and CSF signal fluctuations extracted from T1 image based tissue probability masks from SPM Dartel segmentation; Chang and Glover, 2009; Birn et al., 2014) followed by co-registration of functional to individual structural images and normalization to MNI standard space and final smoothing with a Gaussian Kernel (FWHM 6 mm).

### Data Analysis

Data analysis was based on predefined parcellated regions of interest (ROIs) selected from a functional connectivity atlas (Shirer et al., 2012) delineating the DMN. As explained in the introduction, the DMN undergoes pathology characteristic structural and functional alterations related to cognitive decline in AD. We selected the DMN nodes in medial prefrontal cortex (MPFC), posterior cingulate cortex (PCC), left and right inferior parietal lobes (L-IPL/R-IPL) as well as left and right hippocampi (L-Hipp/R-Hipp). Average BOLD signal fluctuations from all these ROIs were extracted from the individual subjects’ fMRI data and submitted to further analyses.

### Multi-Scale Entropy (MSE) Computation

Entropy was computed for each ROI after averaging the signal fluctuations across all voxels within the respective ROI. We employed sample entropy (SampEn, Richman and Moorman, 2000; Smith et al., 2014) to compute complexity at each scale with pattern matching threshold *r* = 0.2 and pattern length *m* = 2 (Smith et al., 2014; Sokunbi, 2014; Li et al., 2018). Scales were created by coarse sampling of the original time series data into 40 scales; i.e., scale 1 is the original time series and 2 is created by averaging every two consecutive, non-overlapping time points, and similarly for all other scales n the time series was subsampled as averages of n consecutive non-overlapping time points. To identify the scales with reliable entropy values, we statistically compared SampEn at each scale against 0. MSE is the average across all scales considered (Costa et al., 2002; Smith et al., 2014). We further calculated the global DMN complexity as mean MSE across all scales and all nodes. Comparison of entropy between the AD and HC group was performed by two-sample, one-sided *t*-test (significance level *p* < 0.05) on nodal and global DMN level (scales 1–10, 0.625–0.063 Hz).

### Functional Connectivity (FC) Analysis

Seed-to-Seed functional connectivity between every ROI pair was calculated by the Pearson correlation between the average ROI signal fluctuations. For group comparison we considered node-to-node connectivity as well as global DMN connectivity, which was defined as mean FC across all node-to-node correlations (upper-triangle in cross correlation matrix). Comparison of FC between the AD and HC group was performed by two-sample, one-sided *t*-test (significance level *p* < 0.05) on node-to-node and global DMN level.

### Relationship Between MSE, FC, and Cognitive Impairment (MMSE)

First, we calculated the Pearson correlation between DMN FC and DMN-MSE at scales 1–20 (0.625–0.031 Hz) across all participants to elucidate the relationship between network complexity and connectivity. To examine potential associations of MSE with cognitive abilities, partial Pearson correlation coefficients were assessed, corrected for age and gender. Concretely, the global DMN-MSE (MSE averaged across scales 1–10; 0.625–0.063 Hz) was correlated with the MMSE reflecting a general measure of mental health as well as the BNT score mirroring semantic memory retrieval. Unfortunately, no utilizable episodic memory score was available for statistical analysis. Furthermore, we also performed the same correlations with nodal MSE and the neuropsychological tests, for DMN nodes showing significant MSE alteration in the AD group.

## Results

### Functional Connectivity (FC)

**FIGURE 1:**
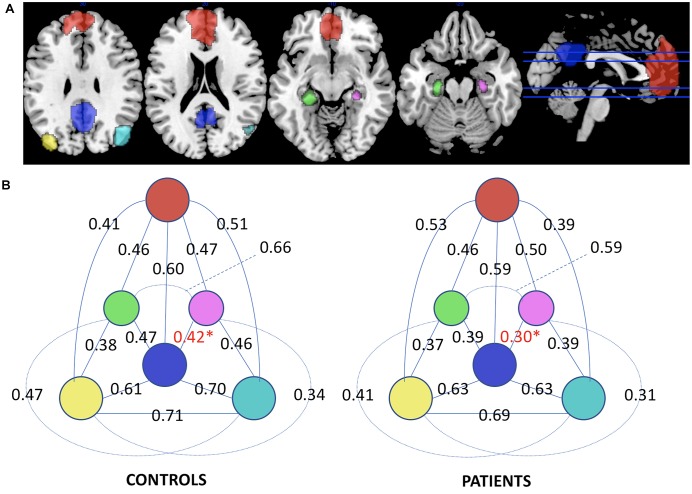
**(A)** Nodes delineating the default mode network. **(B)** Functional Connectivity diagram displaying the correlations between the nodes of the DMN in control and Alzheimer’s disease groups. Significant difference in the R-Hipp/PCC edge for one-sided *t*-test is marked in red and with asterisk (Red: MPFC, blue: PCC, yellow: L-IPL, cyan: R-IPL, green: L-Hipp, purple: R-Hipp).

There were no significant DMN-FC differences between the groups. This was true for whole DMN FC, which is the averaged FC across all node-to-node correlations, as well as for individual node-to-node connections (Figure 1). However, FC to the hippocampal nodes appeared reduced (non-significant, Figure 1). Using one-sided *t*-test assuming that FC is lower in AD than controls we found a significantly reduced connection between PCC and R-Hipp (*t* = 1.90, *p* = 0.034).

### Multi-Scale Entropy (MSE)

The validity analysis for entropy at every scale revealed that for scales 1–20 (0.625–0.031 Hz) we observed a complex behavior of signal fluctuations (Supplementary Figure 1). This is comprehensible since our fMRI comprised 400 volumes and thus coarse sampling at scales 20 and above would produce very short time-series for MSE computation (20 time-points at scale 20) which renders it unreliable. Therefore, for further analysis we restricted entropy values to scales 10 and below to ascertain that entropy values are reliable.

### Network and Nodal MSE Differences

**FIGURE 2:**
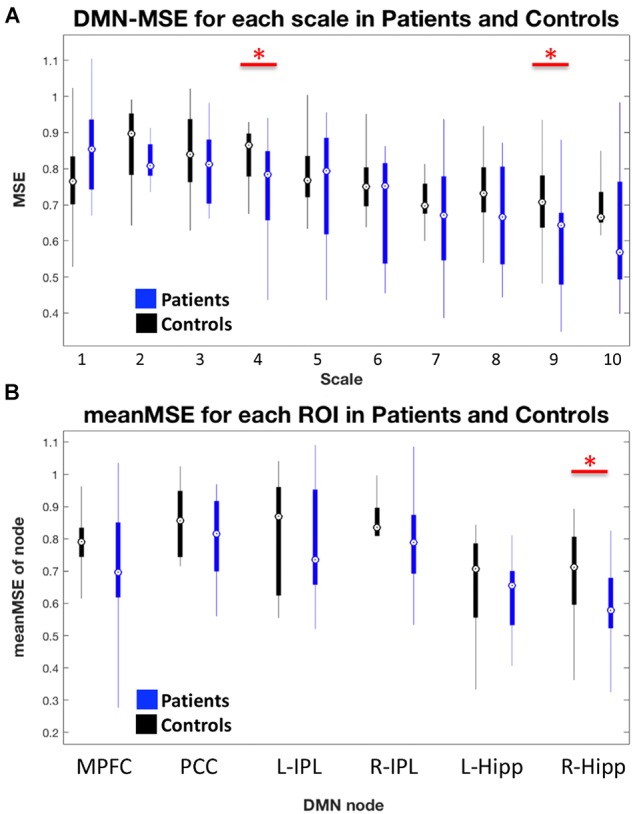
**(A)** Box-plot of entropy values at each scale for whole DMN in HC and AD. **(B)** Box-plot of mean MSE within each node of the DMN. Red horizontal bars with red asterisks above boxes indicate significant differences between patients and controls. Generally, AD had reduced entropy within the DMN and specifically the right hippocampus showed decreased MSE on a nodal level.

We found significantly lower global network level DMN-MSE (*t* = −1.81, one-sided *p* = 0.041) in AD as compared to HC. Figure 2A displays the entropy for DMN at each scale. On a nodal level and across all scales, we found reduced MSE for R-Hipp (*t* = −1.87, *p* = 0.036, Figure 2B) and a trend for MPFC (*t* = −1.42, *p* = 0.083). Detailed analysis of effects for all nodes separated for different scales revealed that the effects for entropy are most pronounced in L-Hipp and R-Hipp which showed consistently reduced entropy for most scales (Supplementary Figure 2). We further found some effects at single scales in MPFC, PCC and R-IPL.

### Correlation Between MSE and FC or MMSE

**FIGURE 3:**
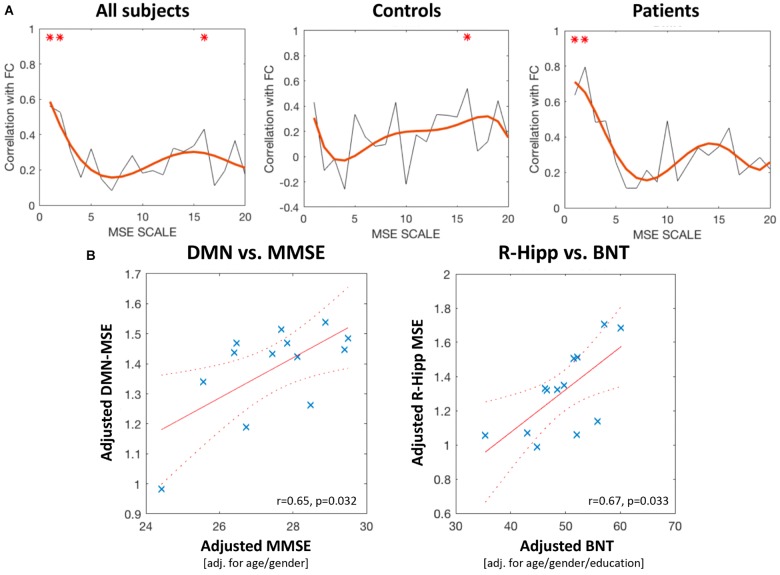
**(A)** Correlation between DMN-FC and DMN-entropy at scales 1–20. The left panel depicts correlations for all subjects, the middle panel for the controls, and the right panel for the Alzheimer’s disease group. Red asterisks mark scales that showed a significant correlation coefficient. **(B)** The left panel shows a scatterplot of the significant positive correlation between global-level DMN entropy (scales 1–10) and MMSE score. The right panel shows the significant positive correlation between R-Hipp entropy (scales 1–10) and BNT score (both panels include patients with AD only).

Across all scales there was a significant positive association between network connectivity and entropy, with significant effects in the AD group in scales 1 and 2 (0.63–0.31 Hz) and in the control group in scale 16 (0.039 Hz, Figure 3A). Moreover, the global DMN-MSE correlated significantly with MMSE in AD patients (*r* = 0.65, *p* = 0.032), but not with the BNT. In contrast, nodal MSE of the R-Hipp showed no association with the MMSE, but with the BNT (*r* = 0.67, *p* = 0.033, Figure 3B). Thus, the lower cognitive abilities of the AD group were associated with a lower overall DMN-MSE, and their declined semantic memory performance was related to a decreased nodal MSE in the R-Hipp. Note that correlational analyses using nodal MSE was only performed with the R-Hipp, because as elucidated in the previous section, this was the only DMN node that yielded a significant MSE change in the AD group across all scales.

## Discussion

In this study, we investigated rs-fMRI signal complexity in DMN nodes in cohorts of mild AD and HC. We additionally compared the outcome of the MSE analysis with the results of the more commonly applied FC analysis.

### Functional Connectivity

First of all, the standard FC analysis did not yield any compelling results. As outlined in the introduction, DMN alterations in AD have been reported repeatedly, yet only moderate to severe disease stages appear to produce reliable results (Buckner et al., 2008; Brier et al., 2012; Cha et al., 2013; Gardini et al., 2015). In the case of our mild AD sample, we merely found indices of reduced FC, mainly between R-Hipp and PCC, and even to a lower magnitude between L-Hipp and PCC, and R-IPL and MPFC (Figure 1). Studies that showed reduced FC of the PCC in AD have raised the hypothesis that cortical hubs such as the PCC are prone to functional deterioration due to their relatively high resting metabolism that is no longer maintained as a consequence of amyloid deposition, which co-occurs in these hubs (Buckner et al., 2005; Sorg et al., 2007; Vlassenko et al., 2010; Franciotti et al., 2013). Notwithstanding the consistency with the literature of the observed FC results indicating lower connectivity in AD, we did not find reduced DMN network FC, nor were the FC differences we observed statistically significant on a robust level.

### Multi-Scale Entropy

In contrast, the AD group showed a lower global DMN-MSE, as compared to HC. This confirmed our hypothesis and implied that the DMN-related signal fluctuation was less complex in AD than HC, which is in line with a previous report about whole brain MSE (Liu et al., 2013). Similarly, Wang et al. (2017) reported lower rs-fMRI-derived complexity in AD than MCI and HC, and Li et al. (2018) found reduced fNIRS-signal complexity in DMN-nodes in AD as compared to controls. By inspecting the DMN-MSE at each scale (Figure 2A), we observed a trend of decreasing entropy with increasing scale in AD. In the HC group, there was an entropy increase from scales 1–4, followed by a decrease from scales 5–10. This distinct pattern between patients and controls might reflect a disturbed local functional integrity in AD, as it has been proposed that smaller scales (i.e., higher frequencies) are related to intra-regional processing, whereas larger scales (i.e., lower frequencies) are thought to be closely associated with inter-regional FC (Vakorin et al., 2011; McDonough and Nashiro, 2014; McIntosh et al., 2014; Wang et al., 2018). The statistical analysis of DMN-MSE revealed a significant MSE-reduction in AD at both scales 4 and 9, supporting that mild AD is characterized by a disturbance of signal complexity (i.e., entropy), which is associated with local and distal information processing. With regard to the mean MSE for each DMN node, we found a significant AD-related decrease in the R-Hipp. This is different to the findings of Wang et al. (2017), who reported decreased complexity in AD in other brain regions (only MPFC of the DMN regions, among others). However, they used permutation entropy analysis (as compared to SampEn in the current study) and the patients with AD they included in the study were cognitively more impaired than those in this study (MMSE 21 vs. 25).

To this point, we can recapitulate that the main results of the current study were a decreased global DMN-MSE in mild AD, a constant reduction of entropy with increasing scales in AD, and a mean-MSE group comparison that showed decreased R-Hipp MSE in AD compared to HC. While these findings fit well into literature and our hypothesis, thoroughly elucidating the MSE differences between AD and HC for each DMN-node for each scale was more challenging.

With reference to Supplementary Figure 2, we identified the MPFC, L-Hipp, and R-Hipp as nodes with decreased MSE in AD in all significant scales. In MPFC and R-Hipp, the differences were located in fine scales, which indicated a local processing disturbance as circumscribed above. In the L-Hipp, significant differences were found at scales 4–8 (except scale 6). We interpreted this as a deterioration of hippocampal processing in general. Our view is supported by studies that described the Hipp (predominantly left-lateralized) as a crucial region for AD not only as an atrophy hot spot, but also with regard to its functional role in episodic and semantic memory, both of which are affected in AD (Burianova et al., 2010; La Joie et al., 2014). Hence, the decreased entropy in the L-Hipp at the smaller scale 4 might be related to the impaired episodic memory encoding. On the other hand, the attenuated entropy found in the larger scales (i.e., 5, 7, and 8) might mirror a disconnection of the L-Hipp within episodic and semantic networks (Allen et al., 2007). Two additional DMN-nodes, namely the PCC and R-IPL, presented a more complicated image. Increased entropy was found at scale 8 in the R-IPL, whereas at scale 2, MSE was reduced. This pattern might have reflected a discrepant change of signal processing at local and network level. As outlined before, the PCC serves as a hub and has been found to be vulnerable to functional change in AD. It occurred thus unexpected to find increased MSE in scale 3, while neighboring scale 4 showed the opposite. There is some evidence from EEG, which, however, is on a different temporal scale, that increased complexity might also be related to cognitive decline in AD (Mizuno et al., 2010). In our view, future research on MSE is needed in order to resolve such findings. Finally, the L-IPL did not evince significant changes at any particular scale.

### Correlation Between Complexity, Connectivity, and Cognitive Decline

In order to investigate the potential link between FC and MSE, we correlated the DMN-MSE at each scale with FC. As can be seen in Figure 3A, correlations were positive throughout the scales. Two peak correlations at scales 1/2 and 16 could be observed. Group-wise correlation uncovered that the second peak in slow frequency entropy was significant only in HC, whereas in AD, this relationship did not reach significance. In contrast, the AD group showed significant associations with higher frequency entropy and FC. Note that from scales 5–20, the corresponding frequencies range from 0.125 to 0.031 Hz which are commonly associated with the low frequency fluctuations of functional connectivity networks (Damoiseaux et al., 2006; De Luca et al., 2006; Shehzad et al., 2009). However, the second peak suggests that not only slow but also fast processes influence FC. Accordingly, even in the higher frequency spectrum of BOLD signal some physiologic information is contained. This aligns with the hypothesis of distinct information contained in high vs. low frequency MSE where the former represents local processing while the latter reflects information transfer between areas and both are critical properties of distributed processing within cortical networks.

Changes of FC as well as entropy measures have been found to accompany cognitive changes related to healthy aging or disease (Jones et al., 2011; Brier et al., 2012; Liu et al., 2013). The positive correlation between global DMN-MSE and the MMSE score in our AD cohort is in accordance with these previous findings and our predictions. This result also corroborated previous studies suggesting that a high signal complexity in general, or particularly in the DMN, is important for cognitive functionality (Sokunbi et al., 2011; Yang et al., 2013). Interestingly, BNT scores correlated positively specifically with the R-Hipp, but not for example with the global DMN-MSE. As discussed above, the hippocampus has been found to play a role in semantic memory retrieval, which endorses our finding. Moreover, La Joie et al. (2014) proposed that the R-Hipp might be a crossroad between episodic and semantic memory networks. Thus, we not only found supporting evidence for the findings of these studies, but additional indication that apart from the well described FC alterations in the R-Hipp in AD, the local information processing is disturbed, which is associated with an impaired memory performance.

### Limitations

The main limitation of the present study is the small sample size (15 AD, 14 HC). This should be taken into account when interpreting the findings. Considering the mild disease state of the AD group, the small MSE and FC effects were statistically underpowered to apply a correction for multiple comparisons. We feel that despite the small sample size, we could show that MSE is sensitive enough to find differences between HC and mild AD. For a first proof of concept study, we highlighted the usefulness of MSE as a new characteristic for BOLD signal fluctuations in AD.

## Conclusion

We found DMN-MSE in AD was reduced as compared to matched controls and that MSE is related to FC as well as cognitive abilities. Our results further suggest that cognitive decline in AD is reflected by decreased signal complexity in network nodes, which might further lead to disrupted DMN-FC. We hypothesize that a loss of nodal (i.e., right hippocampal) signal complexity potentially impairs synchronization across nodes and preempts FC changes. Thus, MSE presents a putative functional marker for cognitive decline that might be more sensitive than FC in mild AD.

## Data Availability

Data can be requested from the corresponding author. The entropy toolbox and all subroutines can be downloaded from the USC Laboratory of Functional MRI Technology website: http://loft-lab.org/index-5.html.

## Author Contributions

MG contributed to the study design, the data acquisition and analysis, and the manuscript writing. DW contributed to the writing and reviewing of the manuscript. TD contributed to the study design. L-OW contributed to the study design and data acquisition. KJ contributed to the data analysis and the manuscript writing.

## Conflict of Interest Statement

The authors declare that the research was conducted in the absence of any commercial or financial relationships that could be construed as a potential conflict of interest.
