# Supplementary figures and images for: Default Mode Network Complexity and Cognitive Decline in Mild Alzheimer’s Disease
Source: Front Neurosci. 2018 Oct 23;12:770. doi: 10.3389/fnins.2018.00770 (PMC6206840; doi:10.3389/fnins.2018.00770)

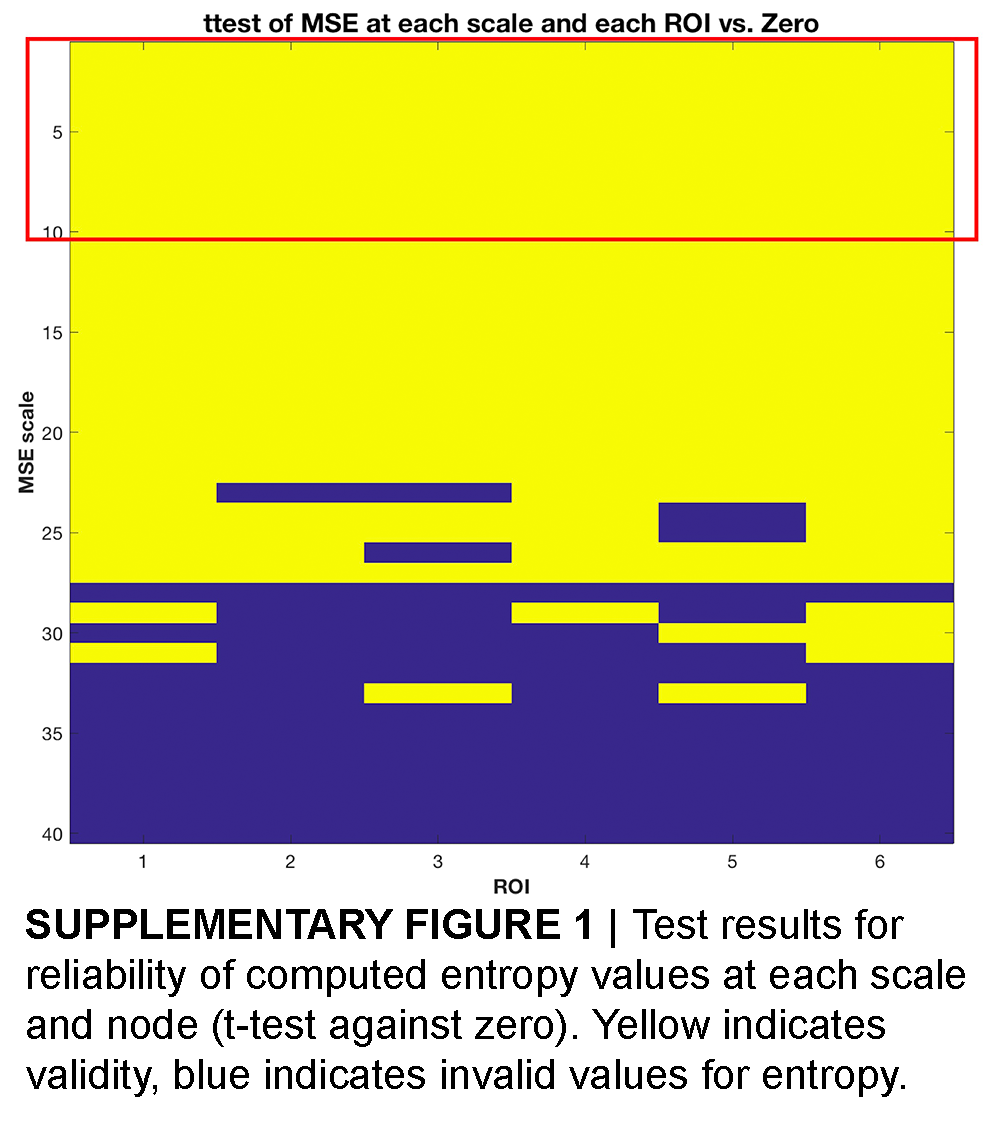

Supplement: Supplementary file 1 [file Image_1.tif]

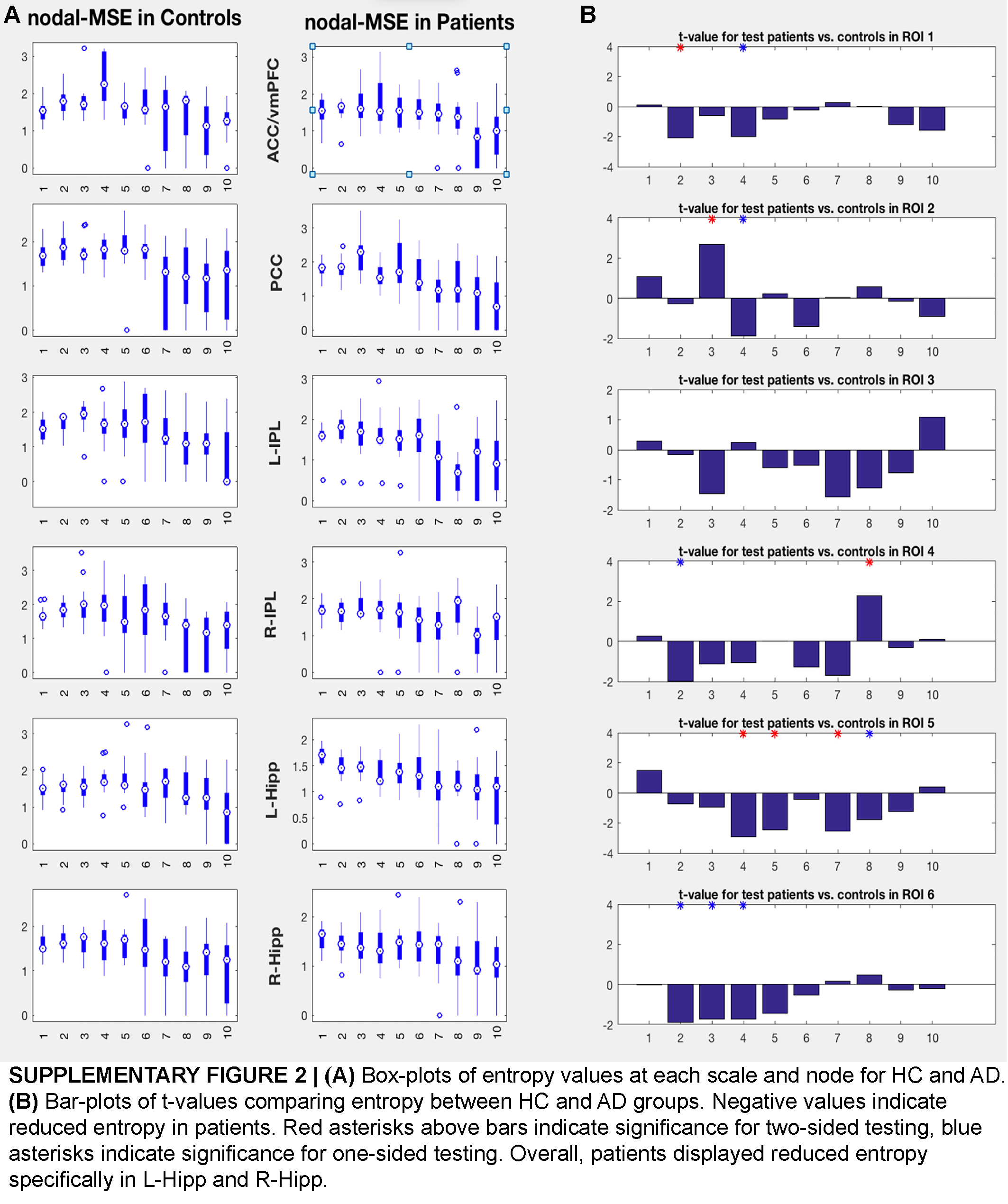

Supplement: Supplementary file 2 [file Image_2.tif]
